# Mr. Merriman's Case of Inversion of the Uterus

**Published:** 1809-01

**Authors:** Samuel Merriman

**Affiliations:** Curson Street, May Fair


### *To the Editors of the Medical and Physical Journal.*

GENTLEMEN,

A Melancholy Case of Inversion of the Uterus has lately been communicated to me, in which the uterus was returned within the vagina, but no attempt was made to re-invert it; consequently the patient is left in a most deplorable situation, deprived of present comfort and enjoyment, and probably doomed in no long time to perish from a serous drain, or some other painful disease which the displacement of so important an organ is likely to produce.

In Ruysh, Bonetus, and other writers, many cases of inverted uterus are recorded, in most or all of which, the patients perished miserably; there can be no doubt that the more judicious management of labors and of the placenta, which the practitioners of the present day very generally adopt, has rendered such accidents less frequent, yet yet the occurrence of them is still occasionally to be lamented. The case above alluded to, proves that when such an event unfortunately takes place, the complaint is not always properly understood, nor properly treated, notwithstanding the rules* which have been very ably laid down for the conduct of the accoucheur under such untoward circumstances.

Several years ago, my friend Dr. Seares and myself had an opportunity of rendering the most essential service to a poor woman, in whom an inversion of the uterus had occurred: with your permission, I will lay the particulars of it before your readers, as an additional proof that the mischief which must necessarily result from an inversion of the uterus, may, by prompt assistance, be prevented.

In January, 1802, Mrs. Edwards, residing in Brick Street, Piccadilly, was delivered of her first child by Mrs. ———, a midwife of considerable practice and experience. The labour was natural, and of no considerable duration. Some little time after the child was born, Mrs. ——— endeavoured by tightening the navel string to extract the placenta, when, (though she asserts that no force was used which could possibly occasion such an event) a very violent pain came on, and the uterus was completely inverted, the placenta remaining attached to it; on this Mr. Sears was immediately called in, who, finding things in this state, desired that I might likewise be sent for.

When I arrived, which was not many minutes after the accident happened, the uterus with the adhering placenta was lying without the *os externum*; blood was flowing profusely, especially from those parts where the placenta was detached; and the woman was in such an exhausted state, that we doubted if she could survive till the uterus should be replaced. We perfectly coincided in opinion respecting the plan to be pursued; and as no time was to be lost, Mr. Sears removed the partially separated placenta, and returned the uterus within the vagina, whilst I was laying bare my arm. I then introduced my hand, carrying the fundus uteri before me, till I had passed my arm quite to the elbow within the vagina; at this moment, I found the fundus uteri, as it were, spring from my hand, and the os uteri began to contract; I therefore cautiously withdrew my hand, and presently found that the hæmorrhage ceased.

Mrs.

* In Dr. Denman's Introduction to the Practice of Midwifery, chap. 15, Sect. 12.

Mrs. Edwards, during the whole operation, was in a state of syncope; but on our giving her some wine and other cordials, she revived, and afterwards recovered perfectly without a single bad symptom. She has since borne several children, and has never found any inconvenience whatever from this alarming and dangerous accident.

The only merit which can be claimed in this case, was for doing immediately, that which was necessary to be done. Had we allowed a very little time longer to elapse before proceeding to reduce the inversion, the patient would probably have sunk beyond recovery, from the profuse hæmorrhage; or, had the hæmorrhage been stopped by the contraction of the uterus, that very contraction would have prevented us from making any impression on the fundus, and the os uteri would have been closely shut against every attempt which we could make to relax it.

The cases which have come to our knowledge, of women surviving this accident, present a most distressing account of mental and bodily sufferings; these sufferings can be prevented by one method, and by one method only; an immediate and absolute determination to re-invert the uterus. A momentary panic in the mind of the operator, may occasion too great a loss of time to allow of his success; for unavailing have proved all endeavours at restoring the parts to their proper situation and use, when once the contraction of the uterus has completely taken place.

I am, &c.

SAMUEL MERRIMAN.

*Curson Street, May Fair.*

